# *In vivo* markers of inflammatory response in recent-onset schizophrenia: a combined study using [^11^C]DPA-713 PET and analysis of CSF and plasma

**DOI:** 10.1038/tp.2016.40

**Published:** 2016-04-12

**Authors:** J M Coughlin, Y Wang, E B Ambinder, R E Ward, I Minn, M Vranesic, P K Kim, C N Ford, C Higgs, L N Hayes, D J Schretlen, R F Dannals, M Kassiou, A Sawa, M G Pomper

**Affiliations:** 1 Department of Psychiatry and Behavioral Sciences, Johns Hopkins Medical Institutions, Baltimore, MD, USA; 2 Russell H. Morgan Department of Radiology and Radiological Science, Johns Hopkins Medical Institutions, Baltimore, MD, USA; 3 School of Chemistry and Faculty of Health Sciences, The University of Sydney, Sydney, NSW, Australia

## Abstract

Several lines of evidence suggest aberrant immune response in schizophrenia, including elevated levels of cytokines. These cytokines are thought to be produced by activated microglia, the innate immune cells of the central nervous system. However, increase in translocator protein 18 kDa (TSPO), a marker of activated glia, has not been found in patients with chronic schizophrenia using second-generation radiotracers and positron emission tomography (PET)-based neuroimaging. In this study we focused on patients with recent onset of schizophrenia (within 5 years of diagnosis). Quantified levels of TSPO in the cortical and subcortical brain regions using the PET-based radiotracer [^11^C]DPA-713 were compared between the patients and healthy controls. Markers of inflammation, including interleukin 6 (IL-6), were assessed in the plasma and cerebrospinal fluid (CSF) in these participants. We observed no significant change in the binding of [^11^C]DPA-713 to TSPO in 12 patients with recent onset of schizophrenia compared with 14 controls. Nevertheless, the patients with recent onset of schizophrenia showed a significant increase in IL-6 in both plasma (*P*<0.001) and CSF (*P*=0.02). The CSF levels of IL-6 were significantly correlated with the levels of IL-6 in plasma within the total study population (*P*<0.001) and in patients with recent onset of schizophrenia alone (*P*=0.03). Our results suggest that increased levels of IL-6 may occur in the absence of changed TSPO PET signal in the brains of medicated patients with recent onset of schizophrenia. Future development of PET-based radiotracers targeting alternative markers of glial activation and immune response may be needed to capture the inflammatory signature present in the brains of patients with early-stage disease.

## Introduction

Epidemiologic, genetic and preclinical studies support a role of early-life infection and/or altered immune response in the etiology of schizophrenia.^1, 2^ Perhaps related to initial insult and immune response, elevated levels of circulating cytokines, many with pro-inflammatory roles, have been repeatedly found in patients with schizophrenia, even in the early stages of disease.^3, 4, 5^ From these findings, a model in which interleukin 6 (IL-6) and other cytokines have detrimental effects on brain maturation and neurotransmission has been proposed.^6, 7, 8^ Furthermore, biological immunotherapies targeting specific cytokines such as IL-6 have been suggested as future treatment strategies.^9^ Nevertheless, it is unclear whether levels of peripheral cytokines reflect levels within the central nervous system (CNS), and the temporal relationship between microglial activation and exaggerated cytokine signaling in schizophrenia has not yet been studied *in vivo*.

Activated microglia and astrocytes in the CNS are the primary sources of cytokines during reactive, inflammatory response, although neurons and endothelial cells may serve as other sources in select conditions within certain signaling cascades.^10, 11, 12^ Quantitative measures of glial cell activation in the different stages of schizophrenia can be probed *in vivo* using radiotracers targeting the translocator protein 18 kDa (TSPO) and positron emission tomography (PET).^13, 14, 15, 16, 17, 18^ Importantly, TSPO expression is greatly increased in activated glial cells in states of brain injury and repair.^14^ Building on the improved pharmacokinetic characteristics of second-generation radiotracers over the index compound [^11^C]PK11195,^18^ two recent *in vivo* PET studies used second-generation radiotracers ([^11^C]DAA1106, [^18^F]FEPPA), and reported no change in binding to TSPO in the brains of patients with chronic schizophrenia.^19, 20^ However, within both study populations, observed variability in regional binding to TSPO suggested that increased expression of TSPO may vary within patients. Binding of [^11^C]DAA1106 in the brains of patients with schizophrenia correlated significantly with positive symptoms as well as duration of illness.^19^ However, the much larger study of patients using [^18^F]FEPPA PET had several methodological advantages and found no difference in regional brain binding of [^18^F]FEPPA in patients with active psychotic symptoms compared with matched, healthy controls.^20^ Finally, a third study by Bloomfield *et al.*^21^ used the second-generation radiotracer [^11^C]PBR28 and PET in patients with chronic schizophrenia as well as those at ultra-high risk for psychosis. There was no difference in binding of [^11^C]PBR28 between groups using standard kinetic analysis based on the two-tissue compartmental model. However, after applying a new kinetic model that accounts for putative binding to vasculature,^22^ and then normalizing regional binding to that of whole brain, a significant increase in [^11^C]PBR28 signal in schizophrenia and in the at-risk state was found in some gray matter regions.^21^ Each of these three studies included at most one or two patients in their first years of schizophrenia and therefore these studies were unable to evaluate whether TSPO in the brain was increased with hypothesized inflammatory processes in the early stages of disease.

Although results from recent PET-based imaging yield conflicting evidence of TSPO PET signal in the brains of patients, robust cytokine release early in the course of schizophrenia was recently supported by meta-analysis of peripheral cytokine abnormalities in patients, including those with first episode of psychosis (FEP).^3, 4, 5^ The meta-analysis by Miller *et al.*^4^ revealed significantly (*P*⩽0.001) higher levels of several peripheral cytokines and cytokine-response modifiers in patients with FEP over controls, including plasma levels of IL-6 (effect size (ES)=1.4), soluble interleukin 2 receptor (ES=1.03), interferon γ (IFNγ, ES=0.57), transforming growth factor β (ES=0.48), IL-1β (ES=0.6), tumor necrosis factor α (TNFα, ES=0.81) and IL-12 (ES=0.98). Meta-analysis of cytokine function in medication-naive FEP also demonstrated significant elevations in peripheral IL-1β, sIL-2r, IL-6 and TNFα.^5^ Some of these same cytokines were also included in two studies of cerebrospinal fluid (CSF) in FEP, although CSF levels of IL-1β were decreased (ES=−0.99, *P*<0.01) and CSF levels of both IL-6 and IL-12 were unchanged in these samples from unmedicated patients with FEP.^4^ In our own published studies of CSF from medicated patients with recent onset of schizophrenia (defined as within the first 5 years of disease) and unmedicated FEP, we showed a consistent trend of increased CSF levels of IL-6 in both patient groups compared with levels in controls.^23, 24^ Still, lack of larger studies of inflammatory markers in CSF of patients with FEP and recent onset of schizophrenia limit the ability to generalize findings of peripheral cytokine abnormalities to the most relevant tissues, namely those within CNS.

We recently demonstrated that PET-based neuroimaging using the second-generation radiotracer [^11^C]DPA-713 provided improved delivery of radiotracer to the brain and properties consistent with improved specific binding to TSPO compared with the first-generation radiotracer, [^11^C]PK11195.^25^ Importantly, we also showed that [^11^C]DPA-713 PET was sufficiently sensitive to detect increases in TSPO in the brains of patients with human immunodeficiency virus-associated dementia^26^ and those with a history of sports-related, repetitive mild traumatic brain injury.^27^

Here we aimed to use [^11^C]DPA-713 and high-resolution PET to compare binding of the radiotracer in the brains of patients with recent onset of schizophrenia to that of healthy controls who were matched in age, gender, highest educational level and body mass index. In parallel, we tested these same individuals for changes in IL-6 levels in CSF and peripheral tissue, along with other markers of peripheral immune response. Through the use of these complementary methods *in vivo*, we sought to characterize better the neuropathological signature of inflammation in patients with recent onset of schizophrenia.

## Materials and methods

### Human subjects

This study was approved by the Johns Hopkins Institutional Review Board. All the participants provided informed consent. Patients with recent onset of schizophrenia (defined as within 5 years of diagnosis) were recruited from the Johns Hopkins Medical Institutions and from hospitals in the surrounding greater Baltimore–Washington, DC area. Inclusion criteria included diagnosis of schizophrenia according to the Diagnostic and Statistical Manual of Mental Disorders-Fourth Edition after completion of diagnostic and clinical assessment administered by a board-certified psychiatrist (JMC). This assessment included the Structured Clinical Interview for Diagnostic and Statistical Manual of Mental Disorders-Fourth Edition Axis I Disorders-Clinician Version,^28^ Scales for the Assessment of Positive and Negative Symptoms (SAPS and SANS)^29^ and the Calgary Depression Scale.^30^ Patients were excluded if they had (1) history of neurological disorder, structural brain abnormality or history of traumatic brain injury with loss of consciousness; (2) history of special education or known learning disability; (3) history of an inflammatory medical condition including but not limited to diabetes, human immunodeficiency virus and/or hepatitis; (4) benzodiazepine use in the past 6 months; (5) substance abuse (for example, cannabis, alcohol) in the previous 6 months or any history of substance dependence except for nicotine; (6) contraindication to participation in magnetic resonance imaging (MRI); or (7) contraindication to participation in PET including pregnancy. Summary severity scores for three dimensions of symptoms (positive, negative and disorganized) were calculated using the sums of global scores collected from the SAPS and SANS.^31^ Chlorpromazine equivalents were calculated based on the report by Andreasen *et al.*^32^

Healthy adults were recruited from flyers posted in the greater Baltimore–Washington, DC area and by word of mouth. All the healthy controls underwent a careful clinical interview and were without history of medical disease or surgery during the previous year. Healthy controls were excluded if they had any of the above-mentioned exclusion criteria or a Diagnostic and Statistical Manual of Mental Disorders-Fourth Edition Axis I disorder. All subjects in both the patient and control groups were also assessed for TSPO (rs6971) genotype as previously described.^26^

### Neuropsychological assessment

All the participants completed a 2-h battery of neuropsychological tests to assess the cognitive function in five domains, namely processing speed, verbal memory, visual memory, ideational fluency and executive function.^33^ Neuropsychological tests (Supplementary Table S1) were administered and scored according to standard instructions by the same study team neuropsychologist who was blind to the clinical and imaging data of all the participants. The factor scores were calculated for each domain after controlling for age, sex, race and premorbid intelligence based on a normative sample.^34, 35^ Premorbid intelligence was estimated using the Hopkins Adult Reading Test.^36^

### Radiotracer synthesis

[^11^C]DPA-713 was synthesized by O-alkylation of its corresponding desmethyl phenolic precursor with [^11^C]methyl triflate in acetone.^37^ [^11^C]Methyl triflate was obtained from dry phase, high specific activity [^11^C]methyl iodide prepared in a General Electric MeI MicroLab (Milwaukee, WI, USA) from [^11^C]carbon dioxide produced in a General Electric PETtrace cyclotron by proton irradiation of a target consisting of oxygen 5% in nitrogen ultra high purity. The radiotracer was purified by reverse-phase high-performance liquid chromatography and formulated by solid phase extraction as a sterile, apyrogenic solution of 14:1 0.9% saline:ethanol. Radiochemical purity at the end of synthesis was >99% with an average specific activity of 303±118 GBq per micromole (8176±3179 mCi per micromole). The radiotracer product met all USP Chapter <823> acceptance criteria.

### Brain imaging acquisition

Each participant was fitted with a thermoplastic facemask to minimize the head motion and underwent radial artery catheter insertion for repeated blood sampling. [^11^C]DPA-713 was delivered via an intravenous bolus injection at the onset of a 90-min dynamic list mode PET acquisition. The average injected dose was 682.3 (±24.1) MBq. Measurement of the arterial plasma input function was conducted as previously described^25^ through the collection of 25–35 blood samples (1 ml) over the course of each 90-min PET scan. An additional eight serial 4 ml samples were collected for radiolabeled metabolite measurements.^38^

The PET scans were acquired using a second-generation High Resolution Research Tomograph scanner (Siemens Healthcare, Knoxville, TN, USA), an LSO-based, dedicated brain PET system with 2.5 mm resolution. The 90 min list mode data were binned into 30 frames (four 15-s, four 30-s, three 1-min, two 2-min, five 4-min and twelve 5-min frames). The data were then reconstructed using the iterative three-dimensional ordered subset expectation maximization algorithm (with six iterations and 16 subsets), with correction for radioactive decay, dead time, attenuation, scatter and randoms.^39^ The attenuation maps were generated from a 6-min transmission scan performed with a ^137^Cs point source before the emission scan. The reconstructed image space consisted of cubic voxels, each 1.22 mm^3^ in size, and spanning dimensions of 31 cm × 31 cm (transaxially) and 25 cm (axially).

All the subjects also underwent brain MRI to facilitate anatomical delineation of regions of interest (ROIs) on brain PET images after PET-MRI co-registration (detailed below). T1-weighted MRIs were obtained on either a 1.5 T Signa Advantage system (GE Medical Systems, Waukesha, WI, USA) or on a Phillips Achieva 3 T scanner (Andover, MA, USA) with a 32-channel head coil to obtain a 1 × 1 × 1 mm three-dimensional MP-RAGE sequence as previously described.^26^

### Measurement of *f*_P_

Plasma free fraction (*f*_P_) was measured using rapid equilibrium dialysis (RED). Plasma samples were isolated by centrifugation from whole blood withdrawn from each participant before radiotracer injection. A 100 μl sample of plasma was spiked with 1 μl of [^3^H]DPA-713 (83 Ci mmol^−1^; Quotient Bioresearch) and added into the sample chamber of Single-Use RED Plate with Inserts (Thermo Scientific, Rockford, IL, USA) with an 8 K molecular-weight cutoff. Then, 300 μl of PBS was added to the buffer chamber and the plate was incubated on an Incubating Microplate Shaker (Fisher Scientific, Waltham, MA, USA) at 37 °C for 4 h. Ten microliters of plasma and 30 μl of PBS were transferred to scintillation vials and mixed with 10 ml of Bio-Safe II (Research Products International, Mount Prospect, IL, USA) counting fluid. All the samples were measured in triplicate. The radioactivity was measured using an LS 6500 Multi-Purpose Scintillation Counter (Beckman Coulter, Pasadena, CA, USA) to obtain the radioactivity in the plasma (*C*_P_) and buffer (*C*_U_). The free, unbound fraction (*f*_P_) was calculated as: *f*_P_=*C*_U_/*C*_P_ × 100 (%).

### PET image analysis

The software package PMOD (v3.3, PMOD Technologies, Zurich, Switzerland) was used in the initial PET image processing and kinetic analysis. Inter-frame motion correction and PET-MRI co-registration were completed as previously described.^27^ Based on the T1-weighted MR images, automated cortical reconstruction and volumetric segmentation were performed with the FreeSurfer image analysis suite (http://surfer.nmr.mgh.harvard.edu/).

Eight ROIs were selected and generated by combining the corresponding subregions provided by FreeSurfer (Supplementary Figure S1). These ROIs included six cortical regions (insula, cingulate, parietal, frontal, temporal and occipital) and two subcortical regions (hippocampus, amygdala). These ROIs were selected to represent the widespread cortical and subcortical involvement found in meta-analyses of structural reduction in brain volume even in patients with first episode of psychosis.^40, 41, 42^ An ROI containing the entire cortical gray matter (GM) was also defined to serve as a normalization factor in the secondary analysis of regional binding relative to GM, as discussed in the previous PET studies using [^11^C]*R*-PK-11195 (ref. 43) and [^11^C]DPA-713.^44^ Finally, total intracranial volume (ICV) was also defined using Freesurfer, for use in secondary analyses exploring the effect of ROI volume normalized to ICV on regional total distribution volume (*V*_T_).

PET time–activity curves (TACs) were generated for all the subjects using the above-mentioned ROI definitions. Based on the time–activity curves obtained, the *V*_T_ within each ROI was estimated using Logan graphical analysis^45^ (t*=30 min) with the metabolite-corrected arterial plasma input function. Following the proposed nomenclature for reversibly binding radioligands,^46^ *V*_T_ represents the ratio of the radioligand concentration in the brain tissue to that in the plasma at equilibrium, which is proportional to the receptor density in the defined ROI. Regional values of GM-normalized *V*_T_ (_GM_*V*_T_) and *V*_T_ corrected for plasma free fraction (*V*_T_/*f*_P_) were also calculated.

### CSF and plasma acquisition

All the participants underwent lumbar puncture after informed consent. All the lumbar puncture procedures were performed at a standardized time (between 1000 h and noon) within the Johns Hopkins Hospital Outpatient Lumbar Puncture Clinic. Upon collection, CSF samples were immediately placed on ice, aliquoted into small volumes and then quickly transferred into a storage freezer at −80 °C. The plasma samples were isolated from whole-blood specimens using centrifugation as described above and were also quickly stored at −80 °C. No samples were thawed more than twice before analysis.

### Measurement of cytokine levels

The plasma and CSF samples were tested for concentrations of IL1β, IFNγ, IL-10 and IL-6 using a V-Plex Custom Human Biomarkers kit (Human Proinflammatory Panel 1), and for TNFα using a Human TNFα kit (MSD (Meso Scale Discovery), Rockville, MD, USA). Each kit provided a 96-well plate pre-coated with anti-cytokine antibodies (four-spot or monospot) at the base of each well. All the reagents were provided in each kit. The control samples were reconstituted in diluent according to the manufacturer's instructions. The human plasma samples were diluted twofold and CSF samples were run undiluted. Sixty microliters of each MSD SULFO-TAG anti-human antibody (anti-IL-1β, anti-IFNγ, anti-IL-6 and anti-IL-10) were combined with the diluent. Sixty microliters of Sulfo-Tag Anti-human TNFα antibody was added to the diluent. Fifty microliters of each prepared control or sample preparation was added to each well and incubated on an orbital shaker for 2 h at room temperature. Each plate was washed three times and 25 μl of detection antibody solution was added to each well. The plates were then incubated on an orbital shaker for 2 h at room temperature, then washed three times and detection buffer was added to each well. Electrochemiluminescence of each MSD SULFO-TAG was captured and quantified using a MESO QuickPlex SQ 120 instrument. The raw data were then analyzed using the Discovery Workbench 4.0 software (MSD) by fitting signal from the control calibration curves to a four-parameter logistic model and then back-fitting the electrochemiluminescence signal from each sample to calculate the unknown concentration. The samples were run in duplicate. Calculated mean concentrations with a percentage coefficient of variance >25% were excluded from the analysis.

The plasma levels of IL-1β were below the levels of detection of this assay for almost all the samples and are not presented. In CSF, the levels of IL-1β, IFNγ, IL-10 and TNFα were often below the levels of detection of this assay. Thus, CSF levels of IL-6 are presented here.

### Statistical analysis

The statistical analyses of the primary PET outcome measure, regional *V*_T_, were performed with multivariate general linear modeling using SPSS Statistics (Version 22.0, IBM, Armonk, NY, USA) so that the effects of several factors could be examined. Specifically, we modeled the *V*_T_ values obtained from the eight selected ROIs based on their relationship to between-subject factors, including cohort (patient or control) and TSPO genotype (C/C: high affinity binder, C/T: mixed affinity binder) as independent fixed factors. Patients with genotype T/T were excluded from this analysis of PET data due to the low affinity of [^11^C]DPA-713 for TSPO in individuals with the T/T genotype. Similar analyses were repeated for regional values *V*_T_/*f*_P_.

All other statistical analyses were performed using R.^47^ Demographic and clinical characteristics of patients vs controls were compared using two sample *t*-tests for continuous variables and Fisher's exact test for categorical variables, with the threshold for significance set to *P*<0.05. Group differences in CSF and plasma inflammatory markers, and in _GM_*V*_T_, were compared using the nonparametric Mann–Whitney *U*-test due to the small sample size. Correlation between CSF and plasma levels of IL-6 was evaluated using Pearson's *r* test. Secondary multivariate analysis using linear regression was used to (1) evaluate effects of clinical characteristics on *V*_T_ in GM and (2) assess the effect of ROI volume normalized to total ICV on *V*_T_ in each ROI, while controlling for genotype in these analyses. Patients with genotype T/T were excluded from the multivariate analysis of PET data. The data were expressed as mean±s.d., unless otherwise noted.

The threshold for significance in all the statistical tests involving regional *V*_T_ was set as *P*<0.006, taking into account multiple comparisons for the eight ROIs using the Bonferroni correction (0.05/8≈0.006). The threshold for significance in all the statistical tests involving the four peripheral markers tested was set as *P*<0.013 (≈0.05/4). Statistical significance was otherwise defined as *P*<0.05, except when otherwise noted.

## Results

### Study population

Demographic characteristics of the study population and clinical characteristics of the patients are presented in Table 1. A total of 14 patients with recent onset of schizophrenia (ages 19–30 years) and 16 healthy control subjects (ages 18–36 years) participated in this study. None of the healthy subjects were using prescribed or over-the-counter medications, with the exception of inclusion of two female participants taking an oral contraceptive. Two of the healthy controls and three of the patients were cigarette smokers. The patients and controls were well matched in age, gender and highest level of education. Two patients were non-adherent with prescribed medications and therefore were not taking antipsychotic medication in the month before the PET scan. One patient was on two second-generation antipsychotic medications and all the other patients were taking antipsychotic monotherapy. The range of chlorpromazine equivalents within the study population was 0–1119.

Two patients and three healthy controls did not participate in the neuropsychological testing. Testing for in the domain of attention was added after the initiation of this study and therefore five additional patients and two additional controls lack neuropsychological performance scores in the domain of attention. The patients showed significant deficits in performance in tests of processing speed, verbal learning and memory, and visuospatial memory compared with healthy controls (*P*<0.05/6=0.008).

### ROI volumes

The quantitative analysis of the MR-based segmentation results for the eight ROIs showed no significant evidence of regional brain atrophy in the patients with recent onset of schizophrenia compared with controls after Bonferroni correction for multiple comparisons (*P*>0.05/8=0.006; Table 2). There were also no significant differences between patients and controls in the volume of the total gray matter and ICV.

### [^11^C]DPA-713 PET imaging

Among the 16 control subjects and 14 patients with recent onset schizophrenia, two healthy controls and two patients were found to have the T/T genotype and were excluded from the PET image analysis. Eight patients had C/C genotype and four patients had C/T genotype. Nine controls had the C/C genotype and five had the C/T genotype.

**Figure 1:**
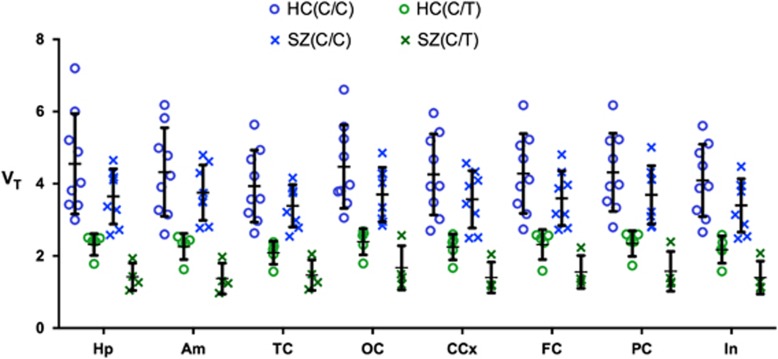
Scatter plot of total distribution volume values (*V*_T_) in the cortical and subcortical regions in patients with recent onset of schizophrenia (SZ) and healthy controls (HC) injected with [^11^C]DPA-713. Individual patient and healthy control *V*_T_ data are shown along with mean values and standard deviation. Using two-way analysis of variance with genetic group (C/C vs C/T) and cohort (SZ vs HC) as independent, fixed factors, regional *V*_T_ values in each of the six cortical regions (insula (In), cingulate (CCx), parietal (PC), frontal (FC), temporal (TC) and occipital (OC)) and two subcortical regions (hippocampus (Hp), amygdala (Am)) did not significantly differ. The threshold for significance after accounting for multiple comparisons was *P*<0.06/8=0.006.

Using two-way analysis of variance with genetic group (C/C vs C/T) and cohort (patients with schizophrenia vs controls) as independent, fixed factors, *V*_T_ values between patients with schizophrenia and controls were not significantly different in all ROIs tested (Figure 1, Supplementary Table S2). Use of both _GM_*V*_T_ (Supplementary Table S3) and *V*_T_ corrected for plasma free fraction (*V*_T_/*f*_P_) did not change these results. Multivariate regression analysis using data from patients and controlling for genotype showed no significant effect of ROI volume normalized to total ICV on *V*_T_ in each of the eight ROIs (*P*>0.05/8=0.006).

### CSF IL-6 levels

Three patients and four controls declined the lumbar puncture for provision of CSF. The concentration of IL-6 in CSF from 11 patients (interquartile range=0.59–1.52 pg ml^−1^, median=0.85 pg ml^−1^) was significantly higher than that in 12 controls (interquartile range=0.44–0.64 pg ml^−1^, median=0.52 pg ml^−1^; *P*=0.02).

### Plasma inflammatory marker levels

**Figure 2:**
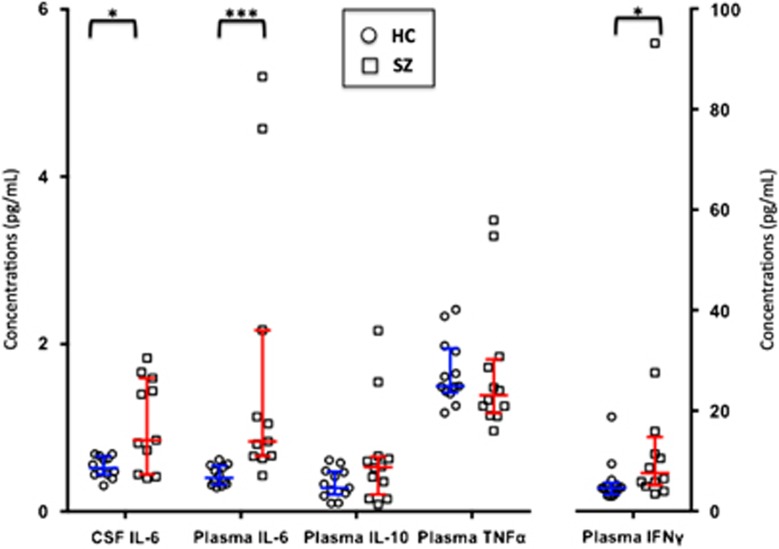
Scatter plot of inflammatory marker levels in the cerebrospinal fluid (CSF) and plasma of patients with recent onset of schizophrenia (SZ) vs healthy controls (HC). Using Mann–Whitney *U*-testing, the concentration of interleukin 6 (IL-6) in CSF from patients with recent onset of schizophrenia was significantly higher than that in controls (*P*=0.02). The plasma concentrations of IL-6 were significantly increased in patients with recent onset of schizophrenia compared with controls (*P*<0.05/4=0.013). Individual patient and healthy control data are shown along with median values and interquartile range. IFNγ, interferon gamma; IL-10, interleukin 10; TNFα, tumor necrosis factor alpha. *P<0.05; ***P<0.001.

The results from CSF IL-6 and plasma markers in patients with recent-onset schizophrenia and controls are shown in Figure 2. Twelve patients and 13 controls provided a plasma sample to test for peripheral markers of inflammation and immune response (IL-6, IFNγ, TNFα, IL-10). After Bonferroni correction for the four peripheral markers tested, the threshold for significance was set to *P*<0.013. The plasma levels of IL-6 were found to be significantly higher in the patients than in the controls (*P*<0.001). Plasma IFNγ was not significantly higher in patients with schizophrenia compared with controls (*P*=0.03). As there were two values of plasma IL-6 levels and one value of plasma IFNγ level in patients where the concentration was measured as five times that of other patients, we ran secondary analysis excluding these potential outliers. The plasma IL-6 and IFNγ levels were significantly higher in patients with recent onset of schizophrenia compared with controls after excluding these values (*P*=0.001 and 0.009, respectively). Plasma levels of the pro-inflammatory marker TNFα and the anti-inflammatory marker IL-10 did not differ between patients and controls.

Finally, the concentration of IL-6 in CSF was found to correlate with IL-6 in plasma within our total study population (*r*=0.70, *P*<0.001) and within the patient cohort alone (*r*=0.63, *P*=0.03).

### Effect of IL-6 on *V*_T_

The secondary multivariate analysis using linear regression revealed no significant effect of both CSF IL-6 and plasma IL-6 on [^11^C]DPA-713 *V*_T_ in GM in the total population after controlling for TSPO genotype (*P*=0.83 and *P*=0.21, respectively). Exploratory analyses of the effects of clinical characteristics, neurocognitive performance and levels of other plasma immune markers on [^11^C]DPA-713 *V*_T_ in GM are presented in Supplementary Table S4. Among these results, there was only one nonsignificant and small, negative effect of chlorpromazine equivalents on *V*_T_ in GM (−0.001, *P*=0.05).

## Discussion

Converging evidence from the epidemiologic, genetic, preclinical and clinical studies of schizophrenia suggest a key role of inflammation and/or altered immune response in schizophrenia, particularly in the early stages of disease.^2, 4, 5, 24, 48^ Activation of microglia, the resident immune cells of the CNS, may not be necessarily deleterious and could be a normal response to independent pathologic processes. Accordingly, the characterization of glial cell activation at the onset and over the early course of disease may inform the temporal relationship to other early pathologic markers and symptomatology. PET-based imaging of TSPO offers a unique opportunity to probe for this marker of activated glial cells (microglia, astrocytes) *in vivo*, based on the increased expression of TSPO by activated glia in states of brain injury or repair.^14, 15, 16, 17, 18, 49^ We recently showed [^11^C]DPA-713 PET allows us to measure increases in TSPO in other neurologic diseases associated with inflammation.^26, 27^

Interestingly, the results from this study are inconsistent with the hypothesized increase of TSPO by activated glia in early schizophrenia. Because of the variability of the regional *V*_T_ values within these patients, we also examined the results using _GM_*V*_T_. Briefly, the use of _GM_*V*_T_ has been shown to be an effective empirical approach^26^ for eliminating genotypic differences while improving the consistency of the data (including intrasubject reproducibility and intersubject agreement among healthy controls). Still, regional _GM_*V*_T_ values did not differ in patients relative to controls. There was also no significant difference in *f*_P_ and *V*_T_/*f*_P_ between patients and controls, which is important as [^11^C]DPA-713 free plasma fractions (~10%) are higher than those reported for other second-generation radiotracers and therefore are more accurately estimated. Finally, as atrophy of the brain has been shown even in patients with FEP,^40, 41, 42, 50^ we also examined the effect of ROI volume normalized to ICV on the *V*_T_ in each respective ROI, but found no effect.

Our results are similar to those from PET-based neuroimaging of patients with chronic schizophrenia, in which use of [^18^F]FEPPA, [^11^C]DAA1106 and [^11^C]PBR28 revealed no change in regional binding (*V*_T_ or BP_ND_) of patients compared with controls.^19, 20, 21^ Bloomfield *et al.*^21^ found higher regional [^11^C]PBR28 PET signal normalized to that of whole brain in patients at ultra-high risk for schizophrenia and in chronic patients using compartmental modeling that also accounted for putative irreversible binding in vasculature (2TCM-1K).^22^ Investigation of this irreversible binding component in [^11^C]DPA-713 PET imaging is ongoing. However, as kinetic modeling of [^11^C]DPA-713 PET data from several healthy controls poorly fits the kinetic model proposed by Rizzo *et al.*^22^ (our unpublished data), and biological evidence for this trapping to vasculature is still lacking, we present outcome variables generated using Logan analysis consistent with previously published literature.^26, 27^ The consistency of our results using *V*_T_ and _GM_*V*_T_ supports the absence of higher [^11^C]DPA-713 PET signal in patients with early-stage disease.

It is important to note that [^11^C]DPA-713 PET signal is only an indirect measure of microglial activation in the living brain. Future translational directions should focus on improved methods for *in vitro* experiments looking for markers of activated microglia directly, in carefully selected postmortem tissue^51, 52^ from early-stage disease. As our patient population showed an increase in both CSF and plasma concentrations of IL-6, a cytokine released by microglia during inflammation, we cannot discount a key role of glial cell activation and immune response in recent-onset schizophrenia. Indeed, an increase in TSPO may be most robust in very early transformation of resting microglia to the activated state and may not necessarily be as marked in chronically active microglia or astrocytes.^53^ On the other hand, we have shown that increased binding of [^11^C]DPA-713 was detectable in the brains of patients with chronic human immunodeficiency virus and those with a history of remote, repeated, sports-related mild traumatic brain injury.^26, 27^ An alternative explanation is that lack of significantly changed TSPO, and perhaps even the downward trend in TSPO PET signal, is linked to dysfunction of mitochondria^54^ on which this protein is expressed, or linked to the more general, aberrant glial cell activity implicated in the pathophysiology of schizophrenia.^55^ These alternatives require further exploration.

One strength of this study is the careful characterization of these patients with early-stage schizophrenia, all of whom have had less than 5 years of disease and treatment. Indeed, even in this early stage of disease, we see predicted, significant deficits in neurocognitive performance. Nevertheless, as mentioned above, we cannot rule out that even the first years of diagnosis are too late along the course of disease to capture the response of activated glia. We must also consider the potential effects of prescribed antipsychotic medication on binding of [^11^C]DPA-713 to TSPO, which may vary by medication. For example, binding of the first-generation radiotracer, [^3^H]PK11195, to TSPO was significantly decreased in an *ex vivo* study of hippocampus from rats treated with sulpiride, thioridazine or risperidone, although binding was significantly increased in this region in rats treated with clozapine.^56^ Although our study population is too small to examine the effects of particular medications on binding to TSPO, chlorpromazine equivalents showed only a very small, insignificant effect on *V*_T_ in GM.

Importantly, even in this population of patients, we still showed significant increases in CSF and plasma levels of IL-6, consistent with results from recent meta-analysis of peripheral cytokine changes in schizophrenia.^3, 4, 5^ To our knowledge, this is the first study to show that an increase in peripheral IL-6 may reflect increased levels of IL-6 in the CNS of patients with schizophrenia. It is important to note that IL-6 has pleotropic activities, and can be secreted by neurons in various states of oxidative stress,^10^ independent of production by microglia in the brain. This point is underscored by the lack of significant increase in several other pro-inflammatory cytokines in the plasma of patients in our study, though IFNγ levels were significantly higher in patients after the removal of an outlier in *post hoc* analyses. It is also possible that the origin of IL-6 in CSF is not from cells of the CNS, but from peripheral inflammatory cells. Peripheral IL-6 may enter the CNS through the choroid plexus, or through a subtle disturbance in the integrity of the blood–brain barrier.^57^ Irrespective of the origin of production, pathologic increase in CNS IL-6 may have detrimental, neuromodulatory effects on the hypothalamic–pituitary–adrenal axis^58^ and a mechanistic role in cognitive deficits seen in schizophrenia.^59, 60^ CNS IL-6 has been proposed as an important mediator of altered synaptic connectivity, brain structure and function in schizophrenia, implicating this cytokine as a potential target for immunotherapy.^9^

By demonstrating lack of significant change in binding of [^11^C]DPA-713 in the brains of patients compared with controls, we support previously noted absent detection of change in TSPO using other second-generation PET-based radiotracers in patients with schizophrenia. Here we extend from those studies with our focus on patients with recent onset of illness who nevertheless have both predicted cognitive deficits and elevated levels of IL-6 in plasma and CSF. Given the interest in further development of interventions targeting cytokine pathways and immune-modulation in early-stage schizophrenia, our results support the development of other PET-based radiotracers targeting alternative markers of glial activation and immune response. On combining PET results with analysis of CSF (and peripheral) tissue from the same individuals *in vivo*, we may be able to elucidate the molecular inflammatory signature in early-stage disease and more meaningfully inform translational application of relevant therapeutic interventions.

## Figures and Tables

**Table 1:** Clinical and demographic characteristics

	*HC* *(*n=*16)*^a^	*SZ* *(*n=*14)*^a^	P^b^
Age (years)	24.9 (4.7)	24.1 (3.1)	0.58
Gender (male)	9 (56%)	11 (79%)	0.26
Years of education	13.0 (2.0)	11.5 (2.3)	0.08
Body mass index	24.9 (4.0)	27.6 (4.0)	0.08
Years of disease		2.2 (1.4)	
CPZ equivalents		474.5 (355.2)	
Atypical antipsychotic (% using)		85.7	
Typical antipsychotic (% using)		14.3	
			
*SAPS/SANS*			
Negative symptom dimension		8.9 (4.1)	
Positive symptom dimension		3.8 (2.5)	
Disorganized symptom dimension		2.9 (2.0)	
Calgary Depression Scale	0.13 (0.34)	3.36 (5.08)	0.03
			
*Neurocognitive domains*			
Processing speed (HC/SZ: 13/12)^c^	95.5 (12.1)	79.4 (13.6)	0.005^d^
Attention (HC/SZ: 9/7)	104.3 (13.9)	92.1 (15.6)	0.12
Verbal learning and memory (HC/SZ: 13/12)	108.3 (14.7)	81.5 (19.1)	<0.001^d^
Visuospatial memory (HC/SZ: 13/12)	99.6 (12.3)	81.3 (16.8)	0.006^d^
Ideational fluency (HC/SZ: 13/12)	105.3 (11.7)	89.5 (15.7)	0.01
Executive function (HC/SZ: 13/12)	102.4 (10.9)	92.7 (17.8)	0.12

Abbreviations: CPZ, chlorpromazine; HC, healthy controls; SAPS, Scale for the Assessment of Positive Symptom; SANS, Scale for the Assessment of Negative Symptom; SZ, patients with recent onset of schizophrenia.

a The data are presented as *N* (%) or mean (s.d.).

b *P*-values for *t*-test or Fisher's exact test as appropriate.

c Numbers of subjects where data are available.

d Indicates significance (*P*<0.008).

**Table 2:** Comparison of measured volumes of each ROI between patients with recent onset schizophrenia and healthy controls who underwent [^11^C]DPA-713 PET

*ROI*	*HC* *(*n=*14)*^a^	*SZ* *(*n=*12)*^a^	P^b^
Hippocampus	8.87 (0.90)	8.41 (0.64)	0.143
Amygdala	3.84 (0.51)	3.36 (0.43)	0.015
Frontal cortex	170.70 (16.43)	157.55 (24.83)	0.134
Temporal cortex	101.61 (11.14)	93.36 (16.46)	0.158
Parietal cortex	111.96 (13.67)	103.90 (19.30)	0.241
Occipital cortex	45.36 (5.22)	44.08 (7.38)	0.620
Cingulate cortex	20.86 (2.58)	18.25 (2.09)	0.009
Insular cortex	29.31 (3.92)	28.89 (4.23)	0.794
Total gray matter	633.20 (63.45)	602.17 (86.13)	0.315
Intracranial volume	1454.10 (157.73)	1434.68 (139.87)	0.742

Abbreviations: HC, healthy controls; PET, positron emission tomography; ROI, region of interest; SZ, patients with recent onset of schizophrenia.

a Regional volumes reported in mm^3^ for the patients and control subjects who underwent PET imaging.

b *P*-values for *t*-tests across the eight selected ROIs, as well as the total gray matter and the total intracranial volume.

Accounting for the eight selected ROIs, the threshold for significance was *P*<0.05/8=0.006.
