# Supplementary material for: In vivo markers of inflammatory response in recent-onset schizophrenia: a combined study using [11C]DPA-713 PET and analysis of CSF and plasma
Source: Transl Psychiatry. 2016 Apr 12;6(4):e777–. doi: 10.1038/tp.2016.40 (PMC4872398; doi:10.1038/tp.2016.40)
Supplement: Supplementary Information [file tp201640x1.doc]

**Supplemental Table S1. Neuropsychological Tests in Each Cognitive Domain**

| **Cognitive Domain** | **Neuropsychological Tests and Measures Used** |
| --- | --- |
| **Processing Speed** | Salthouse Perceptual Comparison Test |
|  | Grooved Pegboard Test |
|  |  |
| **Attention** | Brief Test of Attention |
|  | Digit Span (Forward and Backward) |
|  |  |
| **Ideational Fluency** | CIFA Word Fluency |
|  | CIFA Design Fluency |
|  |  |
| **Verbal Learning and Memory** | Hopkins Verbal Learning Test |
|  |  |
| **Visuospatial Memory** | Brief Visuospatial Memory Test |
|  |  |
| **Executive Function** | Modified Wisconsin Card Sorting Test |

**References**

Benedict, R. H. B. (1997). Brief Visuospatial Memory Test-Revised professional manual. Lutz, FL, Psychological Assessment Resources.

Brandt, J. and R. H. B. Benedict (2001). Hopkins Verbal Learning Test-Revised professional manual. Lutz, FL, Psychological Assessment Resources.

Klove, H. (1963). Clinical Neuropsychology. The medical clinics of North America. F. M. Forester. New York, NY, Saunders. **47:** 1647-1658.

Salthouse, T. A. and R. L. Babcock (1991). "Decomposing Adult Age-Differences in Working Memory." Developmental Psychology **27**(5): 763-776.

Schretlen, D. J. (1997). Brief Test of Attention professional Manual. Lutz, FL, Psychological Assessment Resources.

Schretlen, D. J. (2010). Modified Wisconsin Card Sorting Task professional manual. Lutz, FL, Psychological Assessment Resources

Schretlen, D. J. and T. Vannorsdall (2010). Calibrated Ideational Fluency Assessment professional manual. Lutz, FL., Psychological Assessment Resources.

Wechsler, D. (2008). Wechsler Adult Intelligence Scale Administration and Scoring Manual San Antonio, TX, The Psychological Corporation.

**Supplemental Figure S1. Defined Regions of Interest (ROI)**

**
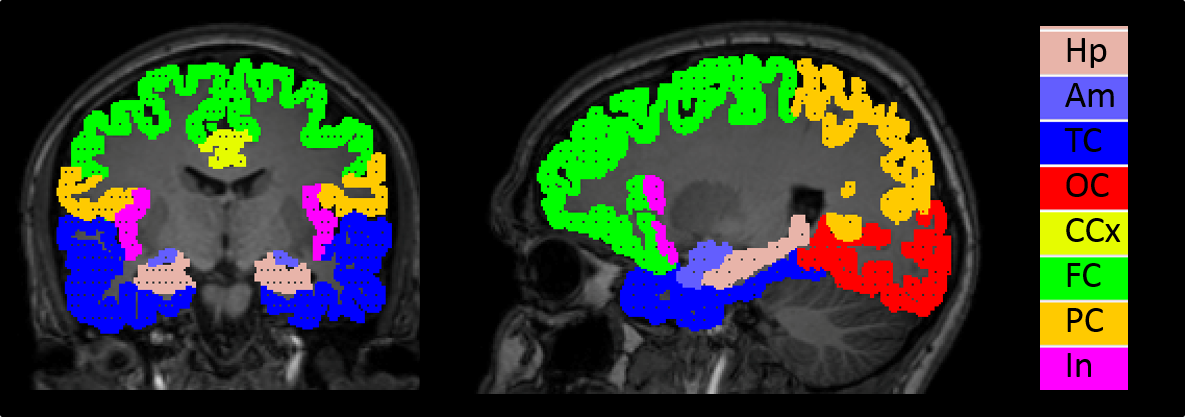
**

This diagram displays an axial and sagittal view of representative magnetic resonance images with color-coded regions of (ROIs), selected and generated by combining the corresponding sub-regions parcellated using FreeSurfer. These ROIs included six cortical regions (insula [In], cingulate [CCx], parietal [PC], frontal [FC], temporal [TC], and occipital [OC]), and two subcortical regions (hippocampus [Hp], amygdala [Am]).

**Supplemental Table S2. Regional [^11^C]DPA-713 Distribution Volume (V_T_) Values**

|  | **C/C** | | **C/T** | |  |
| --- | --- | --- | --- | --- | --- |
|  | **Control**  **(N=9)** | **SZ**  **(N=8)** | **Control**  **(N=5)** | **SZ**  **(N=4)** | ***P*** |
| **Hp** | 4.55 (1.39) | 3.65 (0.77) | 2.32 (0.30) | 1.42 (0.38) | 0.034 |
| **Am** | 4.32 (1.22) | 3.75 (0.77) | 2.26 (0.36) | 1.37 (0.43) | 0.059 |
| **TC** | 3.93 (1.00) | 3.39 (0.59) | 2.09 (0.32) | 1.47 (0.42) | 0.062 |
| **OC** | 4.47 (1.15) | 3.70 (0.75) | 2.39 (0.37) | 1.67 (0.61) | 0.048 |
| **CCx** | 4.26 (1.12) | 3.57 (0.80) | 2.25 (0.36) | 1.40 (0.43) | 0.038 |
| **FC** | 4.28 (1.10) | 3.60 (0.76) | 2.32 (0.42) | 1.56 (0.45) | 0.047 |
| **PC** | 4.32 (1.08) | 3.69 (0.81) | 2.34 (0.36) | 1.57 (0.55) | 0.057 |
| **In** | 4.09 (1.00) | 3.40 (0.74) | 2.17 (0.38) | 1.40 (0.46) | 0.032 |

Regional V_T_ values, listed as mean (standard deviation), are presented for each cohort (patients with recent onset of schizophrenia vs. controls) grouped by genotype (C/C vs. C/T). Using two-way ANOVA with genetic group (C/C vs. C/T) and cohort (patients with schizophrenia vs. controls) as independent, fixed factors, V_T_ values between patients with schizophrenia (SZ) and healthy controls (HC) were not significantly different in all ROIs tested (*P* >0.05/8= 0.006). Regions tested include hippocampus (Hp), amygdala (Am), temporal cortex (TC), occipital cortex (OC), cingulate cortex (CCx), frontal cortex (FC), parietal cortex (PC), and insula (In).

**Supplemental Table S3. Regional [^11^C]DPA-713 Gray Matter-Normalized Distribution Volume (*_GM_V_T_***) Values

|  | **Control**  **(N=14)** | **SZ**  **(N=12)** | ***P*** |
| --- | --- | --- | --- |
| **Hp** | 1.05 (0.07) | 0.99 (0.09) | 0.05 |
| **Am** | 1.01 (0.06) | 1.00 (0.10) | 0.71 |
| **TC** | 0.93 (0.02) | 0.95 (0.06) | 0.12 |
| **OC** | 1.06 (0.04) | 1.05 (0.05) | 0.56 |
| **CCx** | 1.00(0.04) | 0.97 (0.07) | 0.16 |
| **FC** | 1.01 (0.02) | 1.01 (0.02) | 0.56 |
| **PC** | 1.02 (0.02) | 1.02 (0.05) | 0.95 |
| **In** | 0.96 (0.03) | 0.93 (0.04) | 0.05 |

Regional *_GM_V_T_* values, listed as mean (standard deviation), are presented for each cohort (patients with recent onset of schizophrenia vs. controls). Using Mann-Whitney U Testing, *_GM_V_T_* values between patients with schizophrenia (SZ) and healthy controls (HC) were not significantly different in all regions tested (*P* >0.05/8= 0.006). Regions tested include hippocampus (Hp), amygdala (Am), temporal cortex (TC), occipital cortex (OC), cingulate cortex (CCx), frontal cortex (FC), parietal cortex (PC), and insula (In).

**Supplemental Table S4. Effect of patient characteristics on binding (V_T_) in gray matter, controlling for genotype. PET data from individuals of T/T genotype were not included.**

|  | **Estimate** | **P value** |
| --- | --- | --- |
| Genotype (C/C vs C/T) | 1.96 | <0.001 |
| Group (recent onset schizophrenia, control) | -0.68 | 0.04 |
| Sex | 0.13 | 0.72 |
| Age | -0.02 | 0.06 |
| Race  African American vs. Caucasian  Asian vs. Caucasian | -0.10  0.01 | 0.81  1.00 |
| Active smoker | -0.39 | 0.37 |
| Body mass index | -0.06 | 0.13 |
| Years of education | 0.10 | 0.18 |
| Years of disease | -0.19 | 0.21 |
| Calgary Depression Scale | -0.03 | 0.51 |
| CPZ Equivalent | 0.00 | 0.05 |
| Processing Speed | 0.01 | 0.65 |
| Attention | 0.02 | 0.24 |
| Verbal Learning and Memory | 0.01 | 0.48 |
| Visuospatial Memory | 0.01 | 0.40 |
| Ideational Fluency | 0.005 | 0.69 |
| Executive Function | -0.01 | 0.57 |
| Positive symptom dimension | -0.14 | 0.15 |
| Negative symptom dimension | 0.00 | 0.99 |
| Disorganized symptom dimension | -0.10 | 0.35 |
| Plasma IL-6 | -0.18 | 0.21 |
| CSF IL-6 | -0.08 | 0.83 |
| Plasma TNFα | -0.18 | 0.58 |
| Plasma IFNγ | -0.02 | 0.64 |
| Plasma IL-10 | -0.76 | 0.10 |
